# Emerging Therapies in Immune Thrombocytopenia

**DOI:** 10.3390/jcm10051004

**Published:** 2021-03-02

**Authors:** Sylvain Audia, Bernard Bonnotte

**Affiliations:** 1 Service de Médecine Interne et Immunologie Clinique, Centre de Référence Constitutif des Cytopénies Auto-Immunes de l’adulte, Centre Hospitalo-Universitaire Dijon Bourgogne, 21000 Dijon, France; bernard.bonnotte@chu-dijon.fr; 2 Interactions Hôte-Greffon-Tumeur/Ingénierie Cellulaire et Génique, LabEx LipSTIC, INSERM, EFS BFC, UMR1098, Université de Bourgogne Franche-Comté, 21000 Dijon, France

**Keywords:** immune thrombocytopenia, Syk inhibitor, BTK inhibitor, FcRn, desialylation, TPO-RA

## Abstract

Immune thrombocytopenia (ITP) is a rare autoimmune disorder caused by peripheral platelet destruction and inappropriate bone marrow production. The management of ITP is based on the utilization of steroids, intravenous immunoglobulins, rituximab, thrombopoietin receptor agonists (TPO-RAs), immunosuppressants and splenectomy. Recent advances in the understanding of its pathogenesis have opened new fields of therapeutic interventions. The phagocytosis of platelets by splenic macrophages could be inhibited by spleen tyrosine kinase (Syk) or Bruton tyrosine kinase (BTK) inhibitors. The clearance of antiplatelet antibodies could be accelerated by blocking the neonatal Fc receptor (FcRn), while new strategies targeting B cells and/or plasma cells could improve the reduction of pathogenic autoantibodies. The inhibition of the classical complement pathway that participates in platelet destruction also represents a new target. Platelet desialylation has emerged as a new mechanism of platelet destruction in ITP, and the inhibition of neuraminidase could dampen this phenomenon. T cells that support the autoimmune B cell response also represent an interesting target. Beyond the inhibition of the autoimmune response, new TPO-RAs that stimulate platelet production have been developed. The upcoming challenges will be the determination of predictive factors of response to treatments at a patient scale to optimize their management.

## 1. Introduction

Immune thrombocytopenia (ITP) is a rare autoimmune disorder with an incidence of 2.9/100,000 person-years [1,2]. The major complication is bleedings, occurring in 60% of the patients, with serious bleeding in 6%, and intracranial hemorrhage in only 0.4% [1]. These bleedings are favored by a platelet count <20 G/L, the use of anticoagulants or non-steroidal anti-inflammatory drugs [1].

Until now, the management of primary ITP has relied on the utilization of a limited number of drugs, mostly steroids, intravenous immunoglobulins (IVIg), rituximab, thrombopoietin receptor agonists (TPO-RAs) and immunosuppressants, or splenectomy [3]. Treatments are indicated in the case of bleedings, best assessed by a clinical score [4], usually when the platelet count is <20–30 G/L or <50 G/L for patients with other risk factors of bleedings [3]. 

The nature of ITP is also considered for the choice of treatments. Considered as primary in 80% cases, ITP is secondary or associated to another disease (lymphoma, systemic autoimmune diseases, infections or primary immune deficiencies) in around 20% [2]. For example, hydroxychloroquine could be of interest in the case of lupus or presence of antinuclear antibodies [5] and rituximab will be favored in case of ITP associated with lymphoid hemopathy not requiring chemotherapy.

The course of ITP is also considered in treatment algorithm. ITP is defined as newly diagnosed (<3 months after diagnosis), persistent (3–12 months after diagnosis) and chronic (>12 months after diagnosis). Around 70% of adult patients enter the persistent phase and 60% the chronic phase. As remission could spontaneously happen or be obtained with short-course treatments in the first year, irreversible therapy such as splenectomy is excluded before 12 months of evolution. Short-term or sequential therapies are favored in the early phase, as 30% of patients will enter remission within the first 3 months following diagnosis [1,3].

ITP patients are also at increased risk of infections [1], that are partly favored by treatments, highlighting the need for a broader choice of treatments with fewer risks of infectious complications. New therapeutic approaches have been proposed in the last few years and are currently available or under investigation. The purpose of this review is to summarize these new treatments and to situate their mechanisms of action in ITP pathogenesis. 

## 2. ITP Pathogenesis

**Figure 1:**
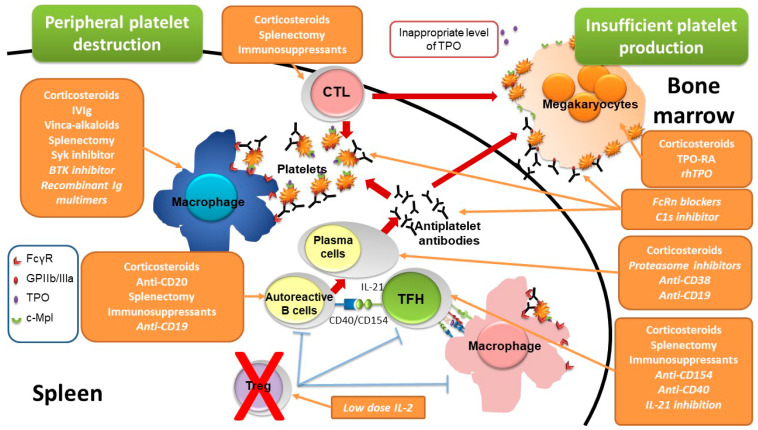
Pathogenesis of immune thrombocytopenia and sites of drug action. Immune thrombocytopenia results from both a peripheral destruction of platelets, mostly occurring in the spleen, and an insufficient bone marrow production. Peripheral platelet destruction is supported by antiplatelet antibodies produced by plasma cells that differentiate from B cells stimulated by T follicular helper cells through the CD40/CD154 axis and IL-21 production. Antiplatelet antibodies target platelet glycoproteins such as GPIIb/IIIa (fibrinogen receptor) leading to platelet destruction by favoring antibody-dependent cellular phagocytosis by macrophages, complement dependent cytotoxicity (CDC) and antibody-dependent cellular cytotoxicity (ADCC). Macrophages also play the role of major antigen-presenting cells capable to stimulate autoreactive T cells. Overall, this autoimmune response is favored by a regulatory T cell deficiency. The insufficient platelet production results from both the autoimmune response targeting megakaryocytes and inappropriate levels of thrombopoietin, the major thrombopoiesis factor. The site of action of the different therapies are reported, drugs not being yet approved or under investigations are mentioned in italic. BTK: Bruton tyrosine kinase, c-Mpl: thrombopoietin receptor, CTL: cytotoxic T lymphocytes, FcγR: IgG Fc receptor, FcRn: neonatal Fc receptor, GPIIb/IIIa: glycoprotein IIb/IIIa, Ig: immunoglobulins, IL: interleukin, IVIg: intravenous immunoglobulins, rhTPO: recombinant human thrombopoietin, Syk: spleen tyrosine kinase, TFH: T follicular helper cells, Treg: regulatory T cells, TPO: thrombopoietin, TPO-RA: thrombopoietin receptor agonist.

ITP is due to an autoimmune peripheral destruction of platelets by their recognition by autoantibodies (Figure 1), targeting different glycoproteins (GPs), mostly GPIIb/IIIa and GPIb/IX, that mediate antibody-dependent cellular phagocytosis (ADCP), antibody-dependent cellular cytotoxicity (ADCC) and complement dependent cytotoxicity (CDC) [6,7]. Opsonized platelets are phagocytosed by splenic macrophages in an Fcγ receptor (FcγR)-dependent mechanism [8]; splenic macrophages also play the role of major antigen-presenting cells in ITP [9]. The desialylation of platelets is another mechanism that could be involved in platelet destruction, desialylated platelets being recognized by the Ashwell–Morell receptor expressed by hepatocytes and being destroyed in the liver in a FcγR-independent mechanism [10]. Antiplatelet antibodies are produced by autoreactive B cells stimulated by T follicular helper cells that provide activation signals through CD40 ligation and interleukin (IL)-21 [11]. Overall, this autoimmune response is favored by a deficiency of regulatory T cells (Treg) [12,13]. There is also insufficient bone marrow production of platelets resulting from both an immune response directed against megakaryocytes [14] but also from inappropriate concentration of thrombopoietin (TPO) [15], the major growth factor of megakaryocytes. 

New therapeutic advances will give the opportunity to target different pathways involved in ITP pathogenesis, either on the peripheral destruction of platelets or the inappropriate bone marrow production (Figure 1). 

## 3. Current Management of ITP 

Treatments of ITP are summarized in Table 1 and their site of actions are depicted in Figure 1.

The cornerstone therapy of ITP remains to be steroids, notably prednisone/prednisolone (1 mg/kg for 2–3 weeks, not exceeding 80 mg/day) or dexamethasone (40 mg/day for 4 days, three cycles maximum), allowing a response in 3–5 days in 85% cases [3]. Whatever the molecule used, there is no long-term effects on ITP evolution [16]. Steroids act by a broad, non-specific, inhibitory effect on the immune response and are associated with numerous complications that preclude their long-term used.

The mechanisms of action of intravenous immunoglobulins (IVIg) are wide and not completely deciphered. The F(ab’)2 portions can neutralize autoantibodies and cytokines or inhibit membrane receptors, while the Fc portion increases the clearance of autoantibodies by saturating the neonatal Fc receptor (FcRn), inhibits the IgG activating receptors (CD16/FcγRIII, CD32A/FcγRIIA, CD64/FcγRI), increases the expression of the inhibitory receptor CD32B/FcγRIIB, and favors the generation of tolerogenic dendritic cells and expands Treg [17]. However, some of these mechanisms of action remain controversial as they have been demonstrated in animal models but not confirmed in humans, such as the upregulation of the inhibitory receptor FcγRIIB on circulating monocytes [18] or on splenic macrophages [19]. Used at 1 g/kg for 1–2 days, they provide a response in up to 90% of the cases [20]. The unresponsiveness occurring in 10–20% of ITP patients could be due to a destruction of platelets not dependent on FcγR, as proposed for anti-GPIb/IX antibodies [21], a hypothesis that is controversial in clinical practice [22,23]. Another explanation could be the predominant involvement of cytotoxic T lymphocytes (CTLs) in the destruction of platelets as demonstrated in an original ITP murine model [24]. 

Rituximab (1000 mg IV, 2 weeks apart or four weekly injections at 375 mg/m^2^) has been proposed in ITP for 20 years, with response rates of 60%, decreasing to 40% and 30% at 1- and 5-year follow-up, respectively [25]. By depleting B cells, anti-CD20 targeting therapies remove precursors of plasma cells that produce pathogenic antibodies [26]. Although side effects were reported in more than half of the 248 patients of the French prospective cohort of patients, around 10% were grade 3/4 [25]. Severe infections occurred in 8.5% but were due to another contributing factor in two thirds of patients. The incidence of severe infection was estimated at 2/100 patients-years and occurred preferentially in non-responder patients. Hypogammaglobulinemia was infrequent, observed in five patients, and related to common variable immunodeficiency, a disease known to be associated with autoimmune cytopenia (AIC), in two cases [25]. 

The combination of dexamethasone (three monthly cycles of 40 mg for 4 days) to rituximab, has been proposed in one study and yielded to a response rate of 64% that dropped to 44% at 1.5-year follow-up [27]. The absence of a control group in this study and the fact that the response rate of rituximab monotherapy is as high as 40% preclude definitive conclusions concerning the clinical relevance of this association [25]. 

A cause of rituximab failure is the emergence of long-lived splenic plasma cells [28] whose survival is maintained by microenvironmental cytokines such as the B cell activating factor (BAFF) [29]. 

Another cause of non-response to rituximab could be the preferential involvement of CTL in the destruction of platelets or megakaryocytes, as supported by the increase in splenic effector memory CTL that produced high amount of interferon-γ and harbor a clonal restriction in ITP patients who did not respond to rituximab [30]. 

To date, there is unfortunately no predictive factors of response to rituximab. Although the response rate seems to be higher in patients with anti-GPIIb/IIIa (75%) antibodies compared to those without (46%) [31], this is not sufficient to determine the use or not of rituximab in clinical practice.

TPO receptor agonists (TPO-RAs), romiplostim (1–10 µg/kg weekly subcutaneous injection) and eltrombopag (25–75 mg/day), increase the production of platelets by megakaryocytes and allow a response in 60–90% of the cases [32,33,34,35]. Overall, their safety is good, with long-term side-effects being represented by thrombosis (6%) and increase in bone marrow reticuline in 1.4 to 6% of cases [32,36,37]. Although their mechanism of action suggests only a suspensive effect, long-term remissions after transient use of TPO-RA have been reported in around 15% of the cases [38,39]. To date, the mechanisms supporting a durable response off therapy remain elusive, but a restoration of Treg functions has been suggested [40]. 

Immunosuppressants, such as mycophenolate mofetil or azathioprine have been usually restricted to patients who failed other therapies, because of the lack of large clinical series [3]. A study is being conducted to assess the potential benefit of mycophenolate mofetil combined to steroids as first line therapy [41].

Despite its high rates of response (66%), splenectomy is less and less performed due to the expansion of medical alternatives and its potential complications [42]. Infections are mostly increased during the 90 days following splenectomy, the long-term risk being similar to the one of non-splenectomized ITP patients [42,43]. While splenectomy is associated with an increased risk of venous thromboembolism [44], the risk of arterial events seems to be similar between splenectomized or non-splenectomized ITP patients [44,45]. Splenectomy allows the removal of the site of platelet destruction but also where the autoimmune response takes place. Again, there is a lack of predictive factors of response, but multiple causes of failure have been described such as the destruction of platelets in the liver or the bone marrow, as assessed by platelet scintigraphy [46], or the survival of autoreactive plasma cells in other niches, notably the bone marrow [47]. 

Despite these different lines of therapy, there are still non-responder patients whose management is challenging, based on the combination of immunosuppressant and TPO-RA, for example [48]. These multi-refractory patients have a higher mortality rate due to both ITP-related bleedings but also to infections favored by immunosuppressive therapies. Thus, new therapies are required to improve the management of ITP patients. 

## 4. New Therapeutic Perspectives

Recently licensed treatments and molecules that are upon clinical investigations in ITP are summarized in Table 1.

### 4.1. New Thrombopoietin Receptor Agonists

Contrary to eltrombopag, avatrombopag and lusutrombopag, two new oral TPO-RA, have no food interactions [49]. Their efficiency seems to be similar to the one of eltrombopag with a response observed in around 70% [50]. Lusutrombopag is for now indicated in thrombocytopenia associated with chronic liver diseases [51], and the study conducted in ITP has terminated earlier due unachievable study objectives (NCT01054443). Avatrombopag has been approved by the FDA and will be soon available in Europe, based on the results of a phase 3 clinical trial conducting in 49 ITP patients, allocated either to avatrombopag or to placebo, and showing a response in 65% cases [49]. 

Hetrombopag showed a response in 58.3 (7/12) and 66.7 (8/12) of ITP patients receiving 5 and 7.5 mg/day, respectively, while only 12.5% (1/8) responded at a dosage of 2.5 mg/day (NCT02403440) [52]. Most of the adverse events were minor with only two cases of grade 3 that were not related to treatment. One limitation for the use of hetrombopag is the decrease in its absorption by alimentation [53].

A human recombinant thrombopoietin (rhTPO) is also used in China and has shown its efficiency in ITP with a response rate of 60% and mild adverse events reported in 13.6% of cases [54]. One advantage of this rhTPO is to be efficient and well tolerated during pregnancy [55], while TPO-RAs are contraindicated. However, the use of TPO-RA during pregnancy (notably for delivery preparation or in case of refractory ITP) has been reported in a short retrospective series of 15 patients (17 pregnancies) that showed a response in 77% of cases without complications among mothers and neonates excepted for one transient neonatal thrombocytosis [56]. Although the use of TPO-RA should not be encouraged in routine clinical practice, it could be interesting transiently to prepare delivery in the absence of other alternatives, or throughout the pregnancy for multi-refractory patients requiring therapy because of bleedings. 

### 4.2. Inhibition of the FcγR Transduction Signal

The phagocytosis of platelets opsonized by auto-antibodies by macrophages required the engagement of FcγR, most particularly FcγRI and III [8]. After the ligation of the immune complexes to FcγR and their cross-linking, there is a phosphorylation by Lyn (a Src family kinase) of the Immunoreceptor Tyrosine-based Activation Motif (ITAM) domains contained in the cytoplasmic portion of the FcγR. This allows the recruitment of the spleen tyrosine kinase (Syk) that phosphorylates multiple substrates leading to the activation of different transduction pathways (Ras/raf/erk, PLC, PKC, etc.). Among the different molecules recruited, the Bruton Tyrosine Kinase (BTK) activates Rac and Rho, involved in the reorganization of the cytoskeleton and required for phagocytosis [57]. Thus, Syk and BTK have become interesting targets to inhibit phagocytosis in ITP.

#### 4.2.1. Spleen Tyrosine Kinase (Syk) Inhibitor

Fostamatinib, a Syk inhibitor, has already been approved for ITP in the USA. The two phase-3 clinical trials included 150 chronic ITP patients who were non-responders to a median of three lines of treatment and allocated either to fostamatinib (100 mg up to 150 b.i.d.) or placebo in a 2:1 randomization. The median duration of ITP was 8.5 years with a median platelet count at inclusion of 16 G/L [58]. An overall response defined as a platelet count ≥50 G/L within the first 12 weeks of treatments was achieved in 43% compared to 14% in the placebo arm. The median time to response was 2 weeks, with most of the patients (83%) achieving a response within 8 weeks. A sustained response, defined as a platelet count ≥50 G/L in at least 4 of the 6 biweekly visits scheduled between weeks 14 and 24 was observed in only 18% [58]. This low stable response rate has raised concerns about the place of fostamatinib in the algorithm of ITP treatments, with some authors arguing that fostamatinib will probably not replace rituximab, TPO-RA or splenectomy [59]. However, post hoc analysis of the phase 3 trials showed higher benefits in terms of platelet response defined as ≥50 G/L (78% vs. 48%) and bleeding events (28% vs. 45%) when fostamatinib was used as second line therapy (*n* = 32), i.e., after steroids or IVIg, compared to third- or later-line (*n* = 113) therapy. Of note, the higher the number of previous lines was, the lesser was the response (64, 52, 36% on third-, fourth- and fifth-line therapy, respectively). Once achieved, the response was maintained no matter the number of lines of treatment previously received [60]. The tolerance to fostamatinib will probably be taken into account to determine its place, as mild to moderate side effects were frequent—the most common being diarrhea (31%) and hypertension (28%) [58]. The occurrence of hypertension is thought to be due to the inhibition of Syk that participates with Vascular Endothelium Growth Factor Receptor (VEGFR) signaling in endothelial cells [61]. This highlights the need for a careful attention to potential vascular remodeling if fostamatinib is used in the long-term. Elevation of liver enzymes has also been observed in 10% of cases. Despite its lower efficiency in multi-treated and refractory patients, fostamatinib will be an option as long-term sustained response could be obtained [62]. The long-term tolerance and efficiency of fostamatinib was also reported in 123 patients enrolled in an open-label-extension study with a median treatment duration of 6.7 months [63], which has confirmed the previous results with an overall response of 44% and a sustained response in 18% after a 28-months follow-up. Similar adverse events were observed (diarrhea, hypertension, nausea, and elevation of liver enzymes).

Beyond the determination of predictive factor of response at a patient level to determine which patients will benefit the most from fostamatinib, one can assume that its association with other treatments not targeting macrophages could be of interest, to improve its efficiency and reduce side effects. Of note, other Syk inhibitors are currently being assessed in ITP (cevidoplenib (SKI-O-703): NCT04056195; HMPL-523: NCT03951623).

#### 4.2.2. Bruton Tyrosine Kinase (BTK) Inhibitors

Apart from Syk, Bruton tyrosine kinase (BTK), which is also engaged in the intracellular transduction signal of FcγR, has become an interesting target. As BTK is expressed by platelets, its inhibition by the pivotal BTK inhibitor ibrutinib showed an inhibition of platelet aggregation that precluded its use during ITP. Nevertheless, ibrutinib showed benefits in AIC related to chronic lymphocytic leukemia (CLL) [64]. In this study, 29/193 patients treated with ibrutinib for CLL had an AIC prior to the initiation of therapy. Eight patients had ITP and five had Evans’ syndrome. Overall, no worsening of AIC was observed; on the contrary, treatments dedicated to AIC were reduced in 42% and discontinued in 25% of the cases. 

Rilzabrutinib, an oral, reversible, covalent molecule highly selective of BTK, has shown a good safety profile in a phase I study [65]. Contrary to ibrutinib, no effect on collagen-induced platelet aggregation was observed with rilzabrutinib, nor bruising or fluctuation of platelet count in healthy volunteers [66]. The preliminary results of a phase 1/2 study conducted in 32 chronic ITP patients treated with rilzabrutinib 400 mg b.i.d. showed promising results [67]. Patients had a median age of 50, with a median duration of ITP of 7.3 years, and were non-responders to a median of six prior therapies. Half of the patients achieved a platelet count ≥30 G/L in the first week. Overall, a platelet count ≥50 G/L was obtained in 42% of the patients, with a persistent response in 71% of the weeks of study. Tolerance was good with transient grade 1/2 adverse events observed in 47% cases, mostly affecting the gastrointestinal tract (diarrhea 29%, nausea 21%). A phase 3 study assessing the efficacy and tolerance of rilzabrutinib in ITP is ongoing (NCT04562766).

### 4.3. Neonatal Fc Receptor (FcRn) Inhibitors

The FcRn is a member of the major histocompatibility complex (MHC) class I family molecules. First identified in placental syncytiotrophoblasts, it is indeed expressed in many cells such as endothelial, epithelial and hematopoietic cells. By its capability to bind to IgG and albumin in acidified endosome, FcRn allows their uptake and their release into the circulation while unbound proteins are degraded into lysosomes. FcRn is thus a homeostatic receptor that extends the half-life of IgG and albumin to 21 days [68]. FcRn inhibition could be of interest in antibody-mediated autoimmune diseases to increase the clearance of pathogenic autoantibodies. Different treatments are being assessed such as monoclonal antibodies (rozanolixizumab [69] or nipocalimab [70]), or IgG_1_ with a modified Fc portion that increases the binding to FcRn (efgartigimod [71]) [72]. In phase 1 studies, a decrease in IgG concentrations, but neither IgA nor IgM was observed, without significant decrease in albumin concentration [69,71]. 

In a recent multicenter, open-labeled phase 2 trial (NCT00718692) including 66 adult ITP patients (median age of 54 years, median ITP duration of 5.8 years, median of 4 prior lines of treatment), rozanolixizumab showed a good tolerance, with only 15 of the 51 adverse events reported being related to treatment, mostly consistent with mild-to-moderate headaches [73]. The response (platelet count ≥50 G/L) was achieved in 66.7% and 54.5% of patients receiving a single subcutaneous infusion at 15 and 20 mg/kg, respectively. With a median platelet count of 15.5 G/L prior to treatment, 50% of the patients achieved a platelet count ≥50 G/L within the first week following infusion [73]. 

In a phase 2 study (NCT03102593) including 38 ITP patients (median age of 41 years, median duration of ITP of 4.8 years, baseline platelet count of 16 G/L), efgartigimod administered intravenously at either 5 or 10 mg/kg showed a stable response (platelet count ≥50 G/L for at least 10 consecutive days) in 38% as compared to none in the placebo arm, with an overall response (platelet ≥50 G/L on at least two occasions) in 46 vs. 25% [74]. There was no safety concern.

Another FcRn inhibitor, batoclimab (NCT04428255) is upon investigation in ITP and two others monoclonal antibodies targeting FcRn are being assessed in autoimmune hemolytic anemia (AIHA) and will probably be of interest in ITP: nipocalimab/M281 (NCT04119050), an aglycosylated IgG_1_, and SYNT001 (NCT03075878), a humanized IgG_4_ that binds to FcRn at both acid and neutral pH [70,75].

### 4.4. Replacement of IVIg by Recombinant Molecules

The mechanisms of action of IVIg are multiple, mostly based on their Fc portion, leading to the saturation of FcγR and FcRn. Although IVIg are highly efficient in ITP, their blood derived origin regularly leads to temporary shortages. To counteract this problem, a recombinant human IgG_1_-based Fc multimer linked to a IgG_2_ hinge sequence (GL-2045/PF-06755347, previously named stradomer) showed promising results in animal models by inhibiting CDC, ADCP and ADCC [76,77]. Notably, GL-2045 reduced thrombocytopenia at a similar level to IVIg in a murine model of passive ITP induced by injection of anti-CD41 antibodies [76]. These results have led to its evaluation in humans (NCT03275740).

Another multimer composed of three human IgG_1_ Fc fragments (CSL730) inhibits ADCC and phagocytosis without activating complement pathways. CSL730 showed its efficiency in animal models of ITP and is currently under investigation in a phase 1 study (NCT04446000). 

A hexameric molecule composed of IgG_1_ and IgG_4_ has been generated to limit the activation of complement, cytokine production and platelet activation but preserving FcγR blocking and showed its capability to prevent ITP in a murine model [78]. 

Other approaches rely on the modification of the glycosylation of IVIg to improve their functions [79]. Hypersialylated IVIg is being assessed in a phase 1 study in ITP (M254, NCT03866577)

Altogether, these studies highlight the growing importance of FcγR targeting in ITP and antibody-mediated autoimmune diseases in general, with specific engineering being developed to improve efficacy and limit adverse events. 

### 4.5. Inhibition of the Classical Complement Pathway

The involvement of complement in ITP pathogenesis, notably via the activation of the classical complement pathway, has been known for many years [80,81] but has not been a major field of therapeutic research until recently. Complement pathway is activated through the ligation of antibodies to platelets, leading to their destruction by CDC, but also increasing their opsonization, thus favoring their phagocytosis by macrophages that expressed complement receptors (notably complement receptor 1 (CR1) that binds to C3b). 

Complement deposition is observed at the platelet surface in more than a half of ITP patients [80,81], and about 30% of ITP patients have at least one complement exploration (C3, C4 or CH50) below the lower range [82]. Moreover, complement activation correlates with disease activity, being higher in patients with an active disease compared to those in partial or complete response [83]. 

Thus, the inhibition of the classical complement pathway has emerged as a potential therapeutic target in ITP. A first proof of concept study showed that the complement activation observed in 47% of 55 sera of ITP patients was abrogated in vitro by a monoclonal antibody targeting C1s (TNT003) [84], which supported clinical trials. Sutimlimab, a humanized form of TNT003, harbors similar properties in vitro, decreasing both C3b deposition and the formation of the membrane attack complex. The interim results of a phase 1 study reported on 12 chronic ITP patients (mean age of 45 years, mean ITP duration of 4.7 years, median baseline platelet count of 19.3 G/L, median number of prior therapies of 6.5) and showed promising results [85]. Sutimlimab was infused weekly for the first two injections, then biweekly for up to 21 weeks. After a 9-week washout period, responder patients could enter a long-term extension treatment phase. An overall response (platelet ≥50 G/L with at least a 2-fold increase in baseline level) was achieved in 42%. This response was durable (more than half of the visits between weeks 5 and 21) in all cases with a complete response (platelet count ≥100 G/L) in 33.3%. The median time to obtain a response was 2 days. No significant adverse events related to sutimlimab were reported. These results strengthen in vitro data but, considering that complement activation is not involved in all ITP patients, it also highlights the crucial need for biomarkers to determine which patients will benefit treatment.

### 4.6. Other B Cell Depleting Therapy Strategies

#### 4.6.1. Combination Therapies

Rituximab has been used for many years in ITP, yielding to a response rate around 50% at 1 year and to 30% at 5 years follow-up [25]. Ex vivo analyses of the spleen of ITP patients [28] have shown that non-response to rituximab was due to the persistence of long-lived plasma cells in an environment rich in BAFF. This was confirmed in a murine model [29] and raised the question of targeting BAFF to improve the response to rituximab. This hypothesis was addressed in a prospective phase 2 trial including 15 non-splenectomized ITP patients (60% persistent and 40% chronic ITP, median age of 50 years, median baseline platelet count of 16 G/L) who received rituximab (intravenous fixed dose of 1 g 2 weeks apart) combined with intravenous belimumab (every 2 weeks for the three first infusions, then every 4 weeks for two other infusions) [86]. Belimumab is a monoclonal antibody licensed in systemic lupus that binds to soluble BAFF, thus inhibiting its ligation to BAFF receptor and TACI (Transmembrane Activator and CAML Interactor) receptors that participate in the regulation of B cells and to their survival. A response (platelet count ≥30 G/L with a least a 2-fold increase from baseline) was achieved in 80%, with a complete response (platelets ≥100 G/L) in one third at 1-year follow-up. No serious adverse events related to treatments were reported; seven minor infections were potentially related to treatments and one patient had a grade 2 serum sickness disease. A decrease by a median of 1 g/L and 0.5 g/L in IgG and IgM concentrations was observed between baseline and 6-month follow-up. Of note, the response to vaccination against pneumococcus performed 2 weeks before treatment was decreased in 30% at 1-year follow-up. Compared to the 40–50% of response to rituximab as monotherapy [87,88], the combination to belimumab seems to yield a better response rate; however, whether this response will be sustained remains to be determined.

#### 4.6.2. Anti-CD20 Targeting Therapies

Humanized monoclonal antibodies have been developed in hematology to limit the induction of antidrug antibodies targeting the murine part or engineered to increase their cytotoxicity. Veltuzumab has shown a good tolerance and efficiency in ITP but is not routinely used in clinical practice [89]. Obinutuzumab, a type II antibody with increased ADCC, is licensed in CLL. Given intravenously at a dosage of 1000 mg at days 1, 8 and 15, it showed a great efficiency in a retrospective cohort of eight patients with CLL-associated AIC. All the patients had a persistent complete response after a median follow-up of 15 months and ITP relapsed in only one patient after 19 months [90]. Whether obinutuzumab will provide a better response and tolerance than rituximab in primary ITP is unknown. 

#### 4.6.3. Plasma Cell Targeting Therapies

Considering the fact that the inefficiency of B cell therapies during ITP is partly due to the persistence of long-lived plasma cells [28], plasma cell-targeting therapies could be of interest in ITP. Bortezomib, a proteasome inhibitor, showed in vitro its capability to deplete long-lived plasma cells and to improve platelet count in a murine model of ITP [91]. This effect was confirmed in a clinical case of ITP [92] and in a short series of AIHA [93] supporting its clinical assessment (NCT03013114). There are, however, concerns due to thrombocytopenia secondary to bortezomib, a frequent complication observed when used to treat myeloma. Another proteasome inhibitor KZR-616 (NCT04039477) was supposed to be investigated in ITP and AIHA, but the study was withdrawn due to the SARS-CoV-2 pandemic [94].

The intravenous anti-CD38 monoclonal IgG_1_ antibody daratumumab has proven its capability to deplete clonal plasma cells in myeloma [95]. Short series have reported on its potential efficiency in post-allogeneic-transplantation-associated thrombocytopenia [96]. Due to its mechanism of action, one concern is the emergence of severe hypogammaglobulinemia and infectious complications. Its safety and efficiency will be assessed in ITP (NCT04703621), as will another anti-CD38 antibody, mezagitamab (TAK-079, NCT04278924), a fully human IgG_1_ that has already shown its safety in phase 1 study, either intravenously and subcutaneously [97]. 

In the same aim, CD19 targeting therapy such as the monoclonal antibody inebilizumab has been developed [98]. As CD19 is slightly expressed on plasma cells, inebilizumab depletes both B cells and plasma cells, which is of interest in antibody-mediated auto-immune diseases. This treatment has been approved for the treatment of neuromyelitis optica spectrum disorders and is under investigations in IgG_4_-related disease (NCT04540497), myasthenia gravis (NCT04524273) and kidney transplant desensitization (NCT04174677). Due to its mechanisms of action, inebilizumab is associated with a decrease in immunoglobulin levels, favoring infections (urinary tracts, nasopharyngitis) in around 20% of patients. Infusion-related reactions were observed in around 12% [98]. If their safety is confirmed, CD19-targeting therapies could be of interest in ITP.

To limit the production of antibodies by B cells, obexelimab (XmAb5871), an anti-CD19 non-depleting B cell monoclonal antibody, has been engineered to have a Fc portion with a high affinity to the inhibitory FcγRIIB. Thus, the Fab portion recognized CD19 expressed by B cells, and its Fc portion engages with the unique FcγR expressed on B cells, FcγRIIB, which is responsible for their inhibition and the reduction in the production of pathogenic antibodies, as demonstrated in vitro with B cells from rheumatoid arthritis patients [99]. Clinical investigations are ongoing in lupus (NCT02725515) and IgG_4_-related disease (NCT02725476) and seem promising but required further confirmations. As ITP is an antibody-mediated disease, such a strategy could be of interest.

### 4.7. T Cell Targeting Therapies

#### 4.7.1. CD40/CD154 Blockade

The CD40/CD154 (CD40L) axis acts as a costimulatory signal at diverse levels. CD40 is expressed by APC that can stimulate autoreactive T cells expressing CD154. CD154 is also expressed by T follicular helper (TFH) cells, which are T cells localized in the germinal centers of lymphoid organs, and which stimulate the proliferation, differentiation, and production of antibodies by B cells. In ITP, splenic TFH cells are expanded and participate in the activation of autoreactive B cells and the production of antiplatelet antibodies [11]. Platelets also express CD154 and can directly activate B cells and the production of antiplatelet antibodies [100]. 

The inhibition of the CD40/CD154 axis by anti-CD154 antibodies has shown a response in around 43% (6/14) of ITP patients treated with ruplizumab (hu5c8), but only 16% (5/31) in patients treated with toralizumab (IDEC-131) [101]. Considering the low rate of response, and the increased rate of thrombosis in animal models [102] and in clinical trials [103] due to the activation of platelet aggregation, the development of CD154 targeting therapies has been hampered. However, it is likely that some ITP patients would specifically benefit from a T cell targeting therapy, but to date there is still a need for biomarkers to identify them. Whether patients with a high level of soluble CD154, which has been observed in around 60% of a cohort of 65 ITP patients [104], would be good candidates for such a therapy remains to be determined. 

To counteract platelet aggregation by CD154-targeting therapies, which is due to the interaction with FcγRIIA receptor expressed on platelets [105], antibodies lacking the Fc portion have been developed. VIB4920, a Fc-deficient CD154 antagonist, has shown its safety in healthy controls and its capability to suppress the humoral response in vitro and in vivo [106]. It is being tested in rheumatoid arthritis (NCT04163991) and Sjogren’s disease (NCT04129164) and could be of interest in ITP.

A Fc-modified anti-CD154 antibody (letolizumab/BMS-986004) has shown its safety in rhesus macaques and its ability to prolong the survival of kidney transplants in this model [107]. Interestingly, the modified Fc portion of this antibody lacks the capability to bind to FcγRIIA expressed on platelets, while retaining the capability to bind to FcRn, thus preserving its prolonged half-life. This monoclonal antibody is also able to increase the proportion of Treg [107] that are known to be deficient in ITP [12,13], while decreasing memory T cells. Thus, one can assume that its use in ITP could promote a short-term response but could also restore tolerance, allowing a sustained long-term response of therapy. The safety and efficacy of letozilumab are under investigation in ITP (NCT02273960). 

Anti-CD40 monoclonal antibodies showed interesting results in monkeys by decreasing the development of germinal centers, thus decreasing antibody production [108]. In humans, iscalimab, a fully human, non-depleting anti-CD40 antibody, showed its safety and capability to inhibit humoral response to vaccination and to inhibit CD154-induced B cell activation, supporting its potential interest in autoimmune diseases [109]. Of note, neither infection nor thrombosis were observed in this phase 1 trial.

A humanized antagonistic anti-CD40 monoclonal antibody (BI655064) has shown its safety in a phase 1 study conducted on 40 healthy volunteers [110]. While the concentration of autoantibodies and the activation of B cells were decreased, there was no significant clinical responses observed in a phase 2 study conducted in a cohort of 67 rheumatoid arthritis patients [111]. BI655064 is currently being assessed in ITP (NCT02009761).

#### 4.7.2. IL-21 Inhibition 

In an attempt to inhibit the action of TFH on B cells, IL-21 has been considered as a new therapeutic target. Indeed, IL-21 is required, together with the interaction between CD154 and CD40, to activate B cells and to promote the production of antibodies [11]. In a murine model of lupus, the blockade of IL-21/IL-21R showed a reduction in B and T cell activation together with an interruption of disease progression [112]. BOS161721, a monoclonal anti-IL-21 IgG_1_, was well tolerated in a phase 1 study. Notably, BOS161721 restores the expression of genes downregulated by IL-21 and involved in Treg homeostasis or T cell activation, suggesting its capability to dampen the autoimmune response and to restore tolerance. The blockade of IL-21/IL-21R is currently investigated in lupus (NCT03371251) and could be of interest in ITP [113]. 

#### 4.7.3. IL-2 Signaling Modulation 

To inhibit effector T cells, daclizumab, a monoclonal antibody targeting CD25, the α chain of IL-2 receptor, has been proposed. Although daclizumab efficiently binds to CD25, a partial response was observed in only one of the eleven ITP patients, thus precluding further investigations [114].

With the aim to restore tolerance, notably by promoting the expansion of Treg or the restoration of their immunosuppressive properties, the utilization of low dose of IL-2 has been assessed. A first report on IL-2 given subcutaneously at 1 million units/day for 5 days, then every 2 weeks for 6 months, in patients with various auto-immune diseases showed an expansion of activated Treg by 2-fold at day 8, with a slight persistent increase overtime by 25%. The treatment did not increase effector T cells, resulting in an increase in the Treg/Teff ratio and a trend to an amelioration of clinical scores [115]. The study is still ongoing and patients with ITP should be enrolled (NCT01988506).

#### 4.7.4. Epigenetic Modulation

Chidamide, a histone deacetylase, is capable of increasing the immunosuppressive functions of Treg and converting effector T cells into Tregs in vitro, leading to platelet correction in a murine ITP model [116], thus supporting the potential interest of molecules modulating epigenetic in ITP (NCT03838354).

In line with this, low dose decitabine (3.5 mg/m^2^ IV day 1–3 every 28 days for three cycles), a demethylating agent used in myelodysplastic syndromes, showed a response in 51% (complete response in 17.7%) of 45 primary ITP patients (median age of 51, disease duration of 23 months, at least three prior lines of treatment) [117]. The median time to respond was 2 days and the response lasted for 6 months in 87% cases and for 18 months in 39%. Adverse events, reported in 29% of cases, were minor, mostly nausea. No predictive factor of response was identified. The mechanisms of action could involve both a boost of Treg functions, a decrease in effector T cells and the promotion of megakaryocyte maturation. These attractive results will need confirmation in larger randomized studies (NCT03252457). 

### 4.8. Inhibition of Platelet Desialylation 

In the last 5 years, a different pathway of platelet destruction has been highlighted in murine ITP models, depending on the specificity of antiplatelet antibodies. As previously known, anti-GPIIb/IIIa antibodies lead to the destruction of platelets by splenic macrophages in a FcγR dependent manner, while anti-GPIb/IX induces the desialylation of platelets, leading to their recognition by the Ashwell–Morell receptor (AMR) and their destruction in the liver [10]. This former mechanism mimics physiological removal of senescent platelets, which lose sialic acid overtime and their binding to the AMR inducing the production of TPO by hepatocytes, thus stimulating platelet production by megakaryocytes [118].

In a cohort of 35 patients, antiplatelet antibodies directed against anti-GPIb/IX were overrepresented in 16 multi-refractory ITP patients. Of note, they had an increase in desialylated platelets and higher levels of TPO [119]. Oseltamivir, a drug that inhibits neuraminidase, the enzyme involved in the desialylation of platelets, was used in combination with an immunosuppressant or a TPO-RA in 10 multi-refractory patients and yielded to a response in two thirds. Interestingly, all the responders had anti-GPIb/IX antibodies. Thus, the inhibition of platelet desialylation could be interesting in patients with isolated anti-GPIb/IX antibodies, although this situation is rare in clinical practice. Moreover, the fact that platelet desialylation is exclusively supported by anti-GPIb/IX has been challenged in humans, as sera containing anti-GPIIb/IIIa antibodies also induced the cleavage of sialic acid on platelets [120]. 

The impact of oseltamivir on platelet count in non-ITP patients has been addressed in a retrospective study involving 343 patients treated with oseltamivir (241 for flu and 102 not infected) and 42 patients with flu but not treated. Oseltamivir induced an increase in platelet count by a mean of 55 G/L, compared to 18 G/L in untreated patients [121]. To determine whether oseltamivir is of clinical relevance in ITP, a phase 1/2 study is ongoing in Canada (NCT03520049).

## 5. Conclusions

Many drugs with new original mechanisms of action will soon be available for ITP and will deeply modify its management. Together with this therapeutic progress, new questions have arisen, such as the place of each drug in the algorithm of ITP management, the safety of their long-term use and the possibility of synergistic combination. Moreover, the various response rates to all these therapies highlight the crucial need for biomarkers to tailor the treatment to patients and optimize their management. 

## Figures and Tables

**Table 1:** Current and emerging therapies of ITP.

Therapy	Targets/Mechanisms of Action	Drugs/Molecules	Development Stage
Steroids	Broad action on immune cells (macrophages, T and B cells) limiting platelet destruction	Prednisone	Licensed
		Dexamethasone	
IVIg	− Decrease in platelet destruction by splenic macrophages		Licensed
	− Increase in antiplatelet antibody clearance by saturation of FcRn		
TPO-RAs	Increase in platelet production from megakaryocytes	Eltrombopag	− Licensed
		Romiplostim	− Licensed
		Avatrombopag	− Licensed (US)
		Lusutrombopag	− Phase 1 (NCT01054443)
		Hetrombopag	− Phase 1 (NCT02403440)
		rhTPO	− Licensed (China)
Immunosuppressants	Inhibition of T and B cells	Azathioprine	− Off-label use
		Mycophenolate mofetil	− Off-label use
		Cyclophosphamide	− Off-label use
Syk inhibitors	Decrease in platelet destruction by inhibition of macrophage phagocytosis	Fostamatinib	Licensed (US)
BTK inhibitors	Decrease in platelet destruction by inhibition of macrophage phagocytosis	- Rilzabrutinib	Phase 3 (NCT04562766)
FcRn blockers	Increase in antiplatelet antibody clearance, thus decreasing peripheral platelet destruction and immune response against megakaryocytes	− Rozanolixizumab	− Phase 3 (NCT00718692)
		− Nipocalimab	− Phase 2 (AIHA, NCT04119050)
		− Efgartigimod	− Phase 2 (NCT03102593)
		− Batoclimab	− Phase 2/3 (NCT04428255)
		− SYNT001	− Phase 2 (AIHA, NCT03075878)
Recombinant immunoglobulin multimers	− Decrease in platelet destruction by splenic macrophages	− GL-2045	− Phase 1 (NCT03275740)
	− Increase in antiplatelet antibody clearance by saturation of FcRn	− CSL730	− Phase 1 (NCT04446000)
		− M254	− Phase 1 (NCT03866577)
Complement inhibitors	Decrease in complement dependent cytotoxicity	Sutimlimab (C1s inhibitor)	Phase 2 (NCT04669600)
B cell depleting therapies	− Decrease in short-lived plasma cells by reducing their B cell precursors, thus decreasing antiplatelet antibodies	Anti-CD20:	
	− Restoration of T cell tolerance	− Rituximab	− Off-label use
		− Veltuzumab	− Off-label use
		− Obinutuzumab	− Phase 3 (lupus, NCT04221477)
Plasma cell targeting therapies	Decrease in short and long-lived plasma cells, thus decreasing antiplatelet antibodies	Proteasome inhibitors:	
		− Bortezomib	− Phase 1 (NCT03013114)
		− KZR-616	− Phase 1 withdrawn (NCT04039477)
		Anti-CD38:	
		− daratumumab	− Phase 2 (NCT04703621)
		− mezagitamab	− Phase 2 (NCT04278924)
B cell and plasma cell targeting therapies	Decrease in short and long-lived plasma cells, thus decreasing antiplatelet antibodies	Anti-CD19:	
		− Inebilizumab	− Phase 3 in autoimmune neurologic disorders (NCT04524273), IgG_4_-RD (NCT04540497)
		− obexelimab	− Phase 3 in lupus (NCT02725515) and IgG_4_-RD (NCT02725476)
T cell targeting therapies	− Inhibition of effector T cells	Anti-CD154:	
	− Inhibition of T follicular helper cells	− ruplizumab	− Stopped
	− Restoration of regulatory T cell function	− toralizumab	− Stopped
		− VIB4920	− Phase 2 in RA (NCT04163991) and SS (NCT04129164)
		− letolizumab	− Phase 1/2(NCT02273960)
		Anti-CD40:	
		− Iscalimab	− Phase 2 in lupus (NCT03656562) and SS (NCT03905525)
		− BI655064	− Phase 1 (NCT02009761)
		IL-21 inhibition:	
		− BOS161721	− Phase 1/2 in lupus (NCT03371251)
		− Low dose IL-2	− Phase 2 (NCT01988506)
Neuraminidase inhibitors	Inhibiting platelet desialylation	Oseltamivir	Phase 1/2 (NCT03520049)
Epigenetic modulation		− Chidamide	− Phase 2 (NCT03838354)
		− Low dose decitabine	− Phase 3 (NCT03252457)
Splenectomy	− Decrease in peripheral platelet destruction by splenic macrophages		
	− Removal of the site of maintenance of the autoimmune response		

BTK: Bruton tyrosine kinase, AIHA: autoimmune hemolytic anemia, IgG_4_-RD: IgG_4_-related disease, IL: interleukin, IVIg: intravenous immunoglobulins, FcRn: neonatal Fc receptor, RA: rheumatoid arthritis, rhTPO: recombinant human thrombopoietin, SS: Sjögren’s syndrome, Syk: spleen tyrosine kinase, TPO-RAs: thrombopoietin receptor agonists.
